# Optimizing Antibiotic Treatment Duration for ESBL-Producing Enterobacteriaceae Bacteremia in ICU: A Multicentric Retrospective Cohort Study

**DOI:** 10.3390/antibiotics14040358

**Published:** 2025-04-01

**Authors:** Camille Le Berre, Maxime Degrendel, Marion Houard, Lucie Benetazzo, Anne Vachée, Hugues Georges, Frederic Wallet, Pierre Patoz, Perrine Bortolotti, Saad Nseir, Pierre-Yves Delannoy, Agnès Meybeck

**Affiliations:** 1 Service de Réanimation et Maladies Infectieuses, Centre Hospitalier de Tourcoing, 135 Rue du Président Coty, 59200 Tourcoing, France; camille.leberre95@gmail.com (C.L.B.); lucie.benetazzo@gmail.com (L.B.); hgeorges@ch-tourcoing.fr (H.G.); pydelannoy@ch-tourcoing.fr (P.-Y.D.); 2 Unité de Recherche, Centre Hospitalier de Tourcoing, 135 Rue du Président Coty, 59200 Tourcoing, France; maxime.degrendel@ch-roubaix.fr (M.D.); saad.nseir@chru-lille.fr (S.N.); 3 Service de Réanimation Médicale, CHRU de Lille, 2 Avenue Oscar Lambret, 59000 Lille, France; marion.houard@chru-lille.fr; 4 Laboratoire de Microbiologie, Centre Hospitalier de Roubaix, 11 Boulevard Lacordaire, 59100 Roubaix, France; anne.vachee@ch-roubaix.fr; 5 Laboratoire de Microbiologie, CHRU de Lille, 2 Avenue Oscar Lambret, 59000 Lille, France; frederic.wallet@chru-lille.fr; 6 Laboratoire de Microbiologie, Centre Hospitalier de Tourcoing, 135 Rue du Président Coty, 59200 Tourcoing, France; ppatoz@ch-tourcoing.fr; 7 Service de Réanimation, Centre Hospitalier de Roubaix, 11 Boulevard Lacordaire, 59100 Roubaix, France; perrine.bortolotti@ch-roubaix.fr

**Keywords:** extended-spectrumbetalactamase-producing Enterobacteriaceae, treatment duration, critical care, bacteremia

## Abstract

Background: The optimal duration of antibiotic treatment for extended-spectrum β-lactamase-producing Enterobacteriaceae (ESBL-E) bloodstream infections (BSI) in intensive care unit (ICU) is not established. We aim to evaluate the frequency and clinical outcomesof a short appropriate antibiotic treatment (≤7 days) (SAT) for ESBL-E BSI acquired in the ICU. We specifically assessed the rate of ESBL-E BSI relapse, and in-ICU mortality. Method: All patients who acquired ESBL-E BSI in three ICU in Northern France between January 2011 and June 2022 were included in a multicenter retrospective cohort study. The factors associated with prescribing short (SAT, ≤7 days) versus long (LAT, >7 days) antibiotic treatment were analyzed. To evaluate the impact of SAT on mortality in the ICU, an estimation was applied using a Cox model with a time-dependent co-variable adjusted by inverse weighting of the propensity score. Results: In total, 379 patients were included. The proportion of patients receiving a SAT was 40% in the entire cohort and 25% in survivors beyond 7 days. In bivariate analysis, the factors associated with prescribing a SAT in survivors were shorter pre-bacteremia ICU stay (*p* = 0.005), lower proportion of chronic renal failure history (*p* = 0.034), cancer (*p* = 0.042), or transplantation (*p* = 0.025), less frequent exposure to carbapenem within 3 months (*p* = 0.015). There was a higher proportion of septic shock (*p* = 0.017) or bacteremia secondary to pneumonia (*p* = 0.003) in the group of survivors receiving a LAT. After adjustment, no difference in survival was found between the two groups (HR: 1.65, 95%CI: 0.91–3.00, *p* = 0.10). Conclusion: In our cohort, one quarter of patients with ESBL-E bacteremia acquired in the ICU surviving beyond 7 days were treated with a SAT. SAT did not appear to affect survival. Patients who could benefit from a SAT need to be better identified.

## 1. Introduction

Extended-spectrum β-lactamase-producing Enterobacteriaceae (ESBL-E) bloodstream infections (BSI) are associated with high rates of treatment failure and mortality, especially when initial probabilistic antimicrobial therapy is inappropriate [1,2]. The standard of care of severe ESBL-E infections is carbapenem. Butits overuse has promoted the emergence of carbapenemase-producing Enterobacteriaceae [3,4]. Reducing antibiotic overuse, including reducing antibiotic treatment duration, is an important target of antibiotic stewardship. Long antibiotic courses favor the emergence of antimicrobial resistance [5]. Multiple studies have evaluated different durations of antibiotic therapy in case of Gram-negative bacteremia to define an equally effective shorter treatment [6]. Recently, several randomized controlled trials showed the non-inferiority of 7 days vs. 14 days antibiotic courses for Gram-negative bacteremia [7,8,9]. These studies were not designed to assess shorter antibiotic course in specific subgroups of patients, especially intensive-care patients. The first two studies included only uncomplicated bacteremia. Patients with hemodynamic instability 48 h prior to randomization were excluded. The third one included both hospitalized patients and outpatients. A recent systematic review and meta-analysis showed that 7 days of antibiotic treatment resulted in a similar outcome as 14 days for patients with controlled Enterobacteriaceae bacteremia [10]. Of note, multidrug-resistant bacteria were involved in 8 to 18% of the cases included in the various studies retained in the meta-analysis. These limitations prevent a strong conclusion on the duration of antibiotic treatment for ESBL-E bacteremia, especially in critically ill patients. The current guidelines state that the duration of therapy should not differ for infections caused by resistant organisms compared to infections caused by susceptible organisms [11,12]. According to French guidelines, the recommended duration of antibiotic treatment for uncomplicated bacteremia is 7 days [13]. We therefore defined short treatment as a duration of antibiotic treatment of ≤7 days. While prior studies suggest a short treatment of 7-day is non-inferior to longer durations in the case of Gram-negative bacteremia, none focus specifically on intensive care unit (ICU) patients. We conducted a multicentric retrospective cohort study in critically ill patients to assess the prescription of short (≤7 days) antibiotic treatment (SAT) versus long (>7 days) antibiotic treatment (LAT) for ESBL-E BSI acquired in the ICU. The retrospective design allowed us to include all the ICU patients suffering from ESBL-E BSI. It avoided recruitment difficulties inherent to prospective studies conducted in the ICU [14]. Finally, we aimed to evaluate the prognosis impact of a reduced antibiotic treatment duration.

## 2. Results

### 2.1. Study Cohort

**Figure 1:**
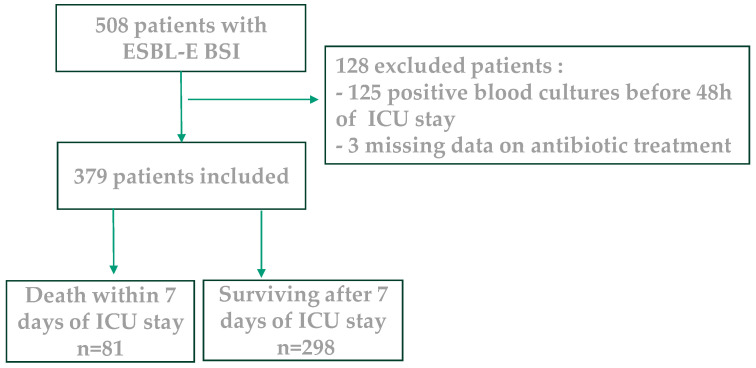
Flow chart of the study population.

Figure 1 represents the flowchart of our study. During the study period, we identified 507 critically ill patients with at least one blood culture yielding ESBL-E. We excluded 128 patients because of ESBL-E BSI onset preceding the ICU admission or missing data about the antibiotic treatment. Finally, 379 patients with ESBL-E BSI acquired in the ICU were included.

Table 1 summarized the characteristics of our patients and clinical data. The patients were mainly male (66%) with a median age of 61 years (51–70). Among our patients, 275 (73%) suffered from at least one comorbidity. The most frequent comorbidity was diabetes, observed in 28% of patients. The majority of our patients entered ICU for medical admission (84%). The median SAPS II value on ICU admission was 50 (38–62). The most common sources of bacteremia were pneumonia (*n* = 212, 56%), a catheter infection (*n* = 102, 27%), an abdominal infection (*n* = 47, 12%), and a urinary tract infection (*n* = 20, 5%). In our cohort, the primary source of infection was pneumonia. The proportion of bacteremia from respiratory source was especially high during the COVID-19 pandemic, reaching 81% of the infections from 2020 to 2022. In the previous years, the proportion of pneumonia was 51.4%.

The most frequently ESBL-E identified were *Klebsiella* sp.(66%), *Enterobacter* sp. (20%), and *Escherichia coli* (9%).

In our cohort, 169 (45%) patients suffered from a septic shock. The mortality rate in the ICU was 49%. Fifty-two patients (14%) experienced relapse. Persistent colonization with multidrug-resistant bacteria was observed in 26% of patients, including colonization with carbapenem-resistant enterobacteriaceae in 6% of patients.

### 2.2. Antibiotic Regimen

**Figure 2:**
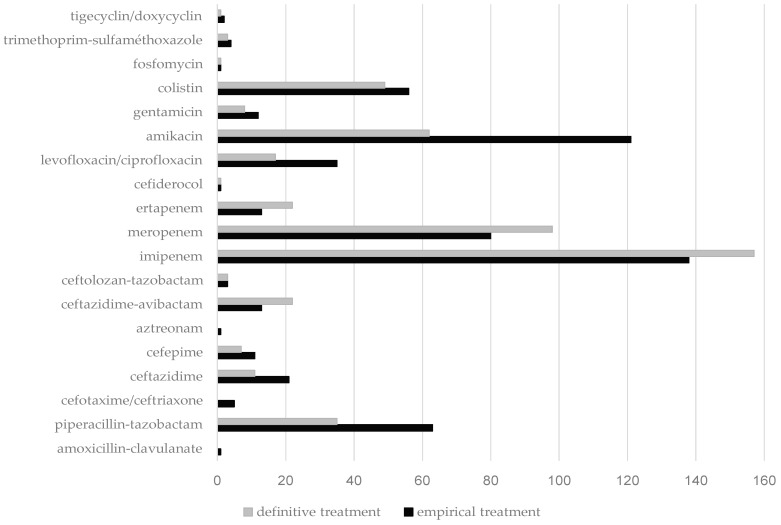
Empirical and definitive antibiotic treatment in patients treated for an ICU-acquired ESBL-E bacteremia.

Figure 2 summarizes the initial empirical and definitive antibiotic prescriptions. Empirical treatment contained carbapenem in 232 of cases (61%). Other betalactams prescribed were mainly piperacillin-tazobactam in 63 patients (17%), ceftazidime in 21 patients (6%), ceftazidime-avibactam in 13 patients (3%), andcefepime in 11 patients (3%). Among our patients, 232 (61%) received an empirical combination therapy, mainly with an aminoglycoside in 136 cases (36%), colistine in 56 cases (15%) or a fluoroquinolone in 35 cases (9%). A total of 293 patients (77%) received an appropriate empirical antibiotic treatment. Of note, the effect of an inappropriate empirical antibiotic therapy on mortality was not significant, OR: 1.35, 95 CI: 0.83–2.2, (*p* = 0.22).

The definitive antibiotic regimen contained carbapenem in 276 cases (73%), piperacillin-tazobactam in 35 cases (9%), ceftazidime-avibactam in 22 patients (6%), ceftazidime in 11 patients (3%), andcefepime in 7 patients (2%). Besides betalactams, the definitive antibiotic regimen contained aminoglycoside (*n* = 71, 19%), colistine (*n* = 49, 13%), or fluoroquinolone (*n* = 17, 4%).

### 2.3. Duration of Antibiotic Treatment and Prognostic Impact

Table 2 summarizes the characteristics and clinical data of all patients included and those who survived beyond 7 days in the ICU based on the duration of appropriate antibiotic treatment received (SAT vs. LAT).

In our cohort, the median duration of appropriate antibiotic treatment was 10 days (5–14) with a maximum prescribed duration of 50 days. A total of 153 patients out of 379 (40%) received SAT. Among the survivors, 48 patients out of 194 (25%) received SAT. The mean duration of bacteremia was similar in both treatment groups (1.46 days in SAT group vs. 1.54 days in LAT group, *p* = 0.41).

In patients surviving beyond 7 days, the factors associated with the prescription of a SAT compared to a LAT were a shorter length of stay in the ICU prior to bacteremia (15 (10–25) vs. 19 (12–32) days, *p* = 0.005), a lower proportion of patients with a history of chronic renal failure (3% vs. 11%, *p* = 0.034) of cancer (5% vs. 14%, *p* = 0.042) or transplantation (0 vs. 6%, *p* = 0.025), less frequent exposure to carbapenem in the last 3 months (13% vs. 30%, *p* = 0.015), and a lower proportion of patients with septic shock (24% in the SAT group vs. 40% in the LAT group, *p* = 0.017). Pneumonia was associated with the prescription of LAT (57% in LAT group vs. 31% in SAT group, *p* = 0.0021). Finally, among survivors, the proportion of appropriate initial antibiotic therapy was higher in the LAT group (83% vs. 62%, *p* = 0.0001).

Regarding the post-bacteremia outcome, no difference was found regarding the duration of vasoactive amine treatment between the 2 groups in survivors beyond 7 days. But survivors receiving LAT had more prolonged mechanical ventilation with a median of 14 days in LAT group vs. 7 days in SAT group (*p* = 0.0001). They experienced more frequently a persistent colonization with multidrug-resistant bacteria (36% vs. 16%, *p* = 0.0021), or a recurrence of ESBL-E bacteremia (26% vs.10%, *p* = 0.03).In the absence of adjustment, the mortality rate in the ICU in the global cohort was 69% in the SAT group compared to 35% in the LAT group (*p* < 0.0001).

After adjusting for immortal time bias and non-random allocation of antibiotic duration, no difference in survival was found between the two groups of antibiotic duration. Indeed, the Cox time-dependent model which allowed control for confounding factors present at treatment initiation and considered treatment duration as a time dependent covariate found HR of 1.65 with, 95% CI: 0.91–3.00 (*p* = 0.10). The sensitivity analysis performed with SAT defined as a duration of antibiotic treatment of ≤10 days found similar results with HR of 1.38 and 95% IC: 0.79–2.42 (*p* = 0.26).

## 3. Discussion

In our cohort of patients suffering from ESBL-E BSI acquired in the ICU, the median duration of antibiotic treatment was 10 days. The proportion of SAT was 40% in the total population, and 25% among survivors beyond 7 days. Our study demonstrated a high mortality among patients who acquired ESBL-E BSI in the ICU. However, after adjustment, the prescription of SAT was not associated with a higher mortality.

An empirical carbapenem-containing regimen was prescribed in 61% of our patients, while almost three quarters of our patients received a definitive carbapenem-containing regimen. For patients with BSI or severe infection due to ESBL-E, the French and international guidelines maintain carbapenem as the preferred treatment [11,12,15]. Prescription of the shortest possible antibiotic therapy will save carbapenems. It will limit the pressure of selection of resistant bacteria and the complications emanating from them [5,16]. The use of carbapenems particularly promotes the emergence of carbapenemase-producing Enterobacteriaceae [3,4]. In our cohort, persistent colonizations with multidrug-resistant bacteria were more frequent in patients who received LAT.

When taking into account only patients surviving beyond 7 days, the proportion of our patients receiving SAT was 25%. The absence of strong recommendations on the duration of treatment for bacteriemia, especially in the most severe patients, leads to disparities in management. A national survey of Canadian infectious diseases and critical-care specialists regarding antibiotic treatment duration for bloodstream infections in critically ill patients showed significant variations amongst clinicians’ recommended durations [17]. However, a majority of clinicians advocated for 10 days of treatment. A similar study was conducted more recently in Australia and New Zealand [18]. Depending on the scenario proposed, one third to almost 60% of the clinicians interviewed suggested the prescription of a short (≤7 days) antibiotic treatment. In these two practice surveys, patient characteristics such as age and comorbidities did not affect the duration of the antibiotic treatment chosen. Similarly, participants reported that they would not change the duration of treatment based on clinical response. The factors considered were the type of infection and the bacteria involved.

In our real-life study, the proportion of SAT appeared lower than that reported in the surveys. The duration of antibiotic treatment seemed to be influenced by patient characteristics. In bivariate analysis, the factors associated with prescribing LAT in survivors beyond 7 days were a history of chronic renal failure, cancer or transplantation, more frequent exposure to carbapenems in the last 3 months, and a longer ICU stay prior to ESBL-E bacteremia. The initial severity and type of infection also seemed to be taken into account in the choice of antibiotic treatment duration. The presence of septic shock at diagnosis and a pulmonary origin of the bacteremia were associated with the prescription of LAT. Inappropriate initial antibiotic therapy did not extend the duration of antibiotic treatment and was even associated with prescription of SAT.

Pulmonary infections are the most frequent infectious complications in the ICU, accounting for up to 50% of ICU-acquired infections [19]. European and French guidelines recommend against a duration longer than 7days of antibiotic therapy for pulmonary infections in most patients [20,21]. The HAS and IDSA recommend not to change the treatment duration for infections caused by a resistant organism [11,12]. However, the association of bacteremia with pneumonia may have motivated the prescription of a longer treatment in our cohort. A recent retrospective study analyzing episodes of nosocomial bacteriaemic pneumonia reported a median duration of antibiotic treatment of 14 days [22]. The presence of bacteremia has been identified as an independent risk factor for mortality in nosocomial pneumonia [23].

An expert opinion recommends that the duration of treatment for uncomplicated bacteremia in the ICU can be matched to that of the source of infection and the causative pathogen [24]. In the absence of a specific risk factor, septic shock, and when the control of the source of infection is appropriate, a short antibiotic treatment of 7 days should be preferred to a longer one. Thus, space is left for a long antibiotic treatment, especially in case of septic shock.

In our study, the mortality rate in the ICU was 49%. This result is concordant with the 44% mortality rate reported by Russo et al. in their cohort of patients with severe sepsis or septic shock due to ESBL-E infection [25]. The reluctance of the ICU physicians to prescribe SAT for ESBL-E BSI can be explained by the potential prognostic impact of the reduction inantibiotic treatment duration. Recently, several randomized controlled trials compared SAT with LAT for the treatment of Gram-negative bacterial infections. Yahav et al. conducted a randomized controlled non-inferiority trial comparing 7 days vs. 14 days of antibiotic treatment in uncomplicated Gram-negative bacteremia [7]. Non-inferiority was demonstrated. The subgroup analysis of resistant pathogens included mainly ESBL-E. Of note, the patients should be afebrile and hemodynamically stable for at least 48 h at baseline. The randomized controlled trial conducted by Molina et al. showed no difference in clinical cure rate of BSI by Enterobacterales between 7-day and 14-day of antibiotic treatments [9]. Only 13% of included patients had sepsis or septic shock and 11% had ESBL-E BSI. Von Dach E. et al. compared a fixed duration of 7 days or CRP-guided antibiotic therapy to 14 days for the treatment of uncomplicated Gram-negative bacteremia [8]. The cure rate was similar in both groups of reduced treatment duration to that observed in the 14-day treatment group. But the median SOFA score was low, and the source of infection was mainly urinary, preventing result extension to critically ill patients and other source of infection. A recent systematic review and meta-analysis showed that 7 days of antibiotic treatment resulted in a similar outcome as 14 days for patients with uncomplicated and controlled Enterobacteriaceae bacteremia, even in the case of hypotension on initial presentation [10]. It should be noted that multidrug-resistant bacteria were involved in only 8 to 18% of the patients included in the various studies. Finally, the recently published BALANCE trial showed that a 7-day antibiotic treatment was non-inferior to a 14-day antibiotic treatment in hospitalized patients with BSI including ICU patients [26]. In the ICU-enrolled patients, the main source of bacteremia was urinary tract infection, and the proportion of resistant organisms was not reported. Moreover, the trial encountered recruitment difficulties, with physicians declining to participate as the main reason for non-randomization. Deviations from protocol were frequent. A pilot randomized trial preparing the BALANCE trial and conducted exclusively in the ICU anticipated these difficulties [14].These limitations prevent solid conclusions from being drawn about the duration of antibiotic treatment in critically ill patients with various source of bacteremia or resistant microorganisms. Our study contributed to bridging existing knowledge gaps concerning the duration of antibiotic treatment of Gram-negative bacteremia. Indeed, it focused on resistant microorganisms, namely, ESBL-E, and on ICU-acquired bacteremia. In our retrospective cohort study including 379 patients treated for ESBL-E BSI acquired in the ICU, after adjustment of the immortality time bias and the biases related to non-random allocation of antibiotic treatment duration, no difference in survival was found between the SAT and LAT groups.

Our study has several limitations. It is a multicenter retrospective observational study. Data collection, although multicentric, was carried out in three ICUs in Northern France, potentially applying an antibiotic strategy different from that used in other regions of the world. This reduces its external validity. However, prescriptions of carbapenems were in accordance with national and international guidelines. The median duration of antibiotic treatment was consistent with that stated by several surveys conducted in different regions of the world. Conversion from IV to oral antibiotic therapy was not recorded. Its impact on in-ICU mortality could not be assessed. Our study, like any observational study assessing the impact of duration of treatment on patient outcomes, is limited by survivor bias (patients must survive long enough to be classified as receiving longer treatment) and indication bias (clinicians select sicker patients to receive longer-duration treatment). The immortal time bias was addressed by the use of a Cox model. The application of a weighted inverse propensity score (IPTW) method reduced the bias related to non-random allocation of treatment duration. Despite our efforts to correct these biases, relevant data to take into account some confounding factors were missing. The criteria used by clinicians to make decisions, differences in physician prescribing behavior, variations in the ICU protocols or infection control measures were not collected. Finally, despite a large sample size, a lack of statistical power in the model cannot be ruled out. However, our results are consistent with the literature.Despite its limitations, our study remains an alternative to randomized trials. Indeed, randomized trials concerning the duration of treatment in the ICUface with recruitment difficulties and frequent deviations from the protocol. Large studies would be needed to assess the prognostic impact of duration of treatment in critically ill patients with ESBL-E BSI.

## 4. Patients and Methods

### 4.1. Setting and Patients

We conducted a multicenter retrospective cohort study in the ICU of two general hospitals (Roubaix and Tourcoing Hospitals) and one academic hospital (Lille Hospital) in the North of France from 1 January 2011 to1 June 2022.

Inclusion criteria:

- Adult patients;
- Admitted to one of the participating ICUs during the study period;
- With at least one blood culture yielding ESBL-E after 48 h of ICU stay.

Exclusion criteria:

- Positive blood culture before 48 h of ICU stay;
- Missing data on antibiotic treatment.

Patients with multiple episodes were included only once in the analysis, and subsequent episodes were considered recurrences or re-infections. Cases were identified using a laboratory and ICU data base query. Enterobacteriaceae identifications, in vitroantimicrobial susceptibility testing and ESBL diagnosis were performed with a Vitek 2 system (bioMérieux^®^, Marcy l’Etoile, France) following the European Committee on Antimicrobial Susceptibility Testing (EUCAST) guidelines [27].

### 4.2. Ethical Approval and Consent to Participate

Our study was carried out in accordance with national guidelines concerning a retrospective observational study conducted on collected data (article R.1121-1-1, Décret No. 2017-884 du 9 May 2017). It obtained approval from the local ethics committee of Dron Hospital and from the National Data Processing and freedoms Commission (No. CHT/URC/2023/06) on 10 June 2023.

### 4.3. Data Collection

The following data were collected on ICU admission: demographic data, indication(s) of ICU admission, underlying clinical conditions, factors of immunodeficiency, and severity of illness. The underlying clinical conditions were classified following McCabe and Jackson criteria [28]. Neoplasia, neutropenia, treatment with glucocorticosteroids, and/or other immunosuppressive therapy, and AIDS were defined as factors of immunodeficiency. Severity of illness was assessed by the Simplified Acute Physiology Score II (SAPS II) and Sepsis-related Organ Failure Assessment (SOFA) score [29,30].

The following data were collected on ESBL-E BSI onset: prior antimicrobial therapy within three months, duration of hospital and ICU stay, severity of illness, presence of shock, and ongoing antibiotic treatment. Empirical antibiotic treatment was defined as the prescription of antibiotics before culture results were available. It was considered appropriate when the isolated bacteria weresusceptible in vitro to at least one of the initial administered antibiotics according to the EUCAST breakpoints. The timing of empirical antibiotic treatment was considered appropriate when it was started during the first 24 h after BSI onset. No specific protocol for antibiotic treatment was applied and the choice of empirical and documented antibiotic treatment wasleft to the clinician in charge of the patient.

### 4.4. Objectives and Definitions

The main objective of our study was to assess the antibiotic duration in patients suffering from ICU-acquired ESBL-E BSI. The primary judgement criterion was the proportion of short antibiotic treatment (SAT), defined as a duration of appropriate antibiotic treatment of 7 days or less. The secondary objectives were to determine factors associated with the prescription of SAT and to assess its prognostic impact. To evaluate the prognostic impact of the antibiotic duration, the rate of ESBL-E BSI relapse, emergence of resistance, *C. difficile* colitis, and in-ICU mortality were recorded.

### 4.5. Statistical Analysis

Continuous variables were expressed as mean values ± standard deviation or as median (interquartile range), depending on the normality of their distribution. They were compared using Student’s test or the Mann–Whitney U test, as appropriate. Categorical variables were expressed as percentages. They were compared using the chi-square test and Fisher’s test when appropriate. Differences between groups were considered to be significant for variables yielding a *p* value ≤ 0.05.

To address the immortal time bias, the treatment duration group was considered a time-dependent covariate within a Cox model. After 7 days of antibiotic treatment, the patients were transferred to the long (>7 days) antibiotic treatment (LAT) group until the end of follow-up. To correct the bias related to non-random allocation of treatment duration, we used a weighted inverse propensity score (IPTW) method. The absence of randomized treatment allocation could introduce confounding bias. The IPTW method aims to simulate a randomized trial by weighting observations according to the probability of receiving the treatment actually observed, thus minimizing the impact of confounding factors. We performed a logistic regression to identify factors associated with short antibiotic treatment (SAT). The covariates included in the model were age, the sum of comorbidities (calculated from the presence of chronic heart failure, diabetes, COPD, cirrhosis, chronic alcoholism, chronic renal failure, solid cancer, malignant hemopathy, asplenia, HIV infection, solid organ transplantation, and neutropenia), and the severity score at ICU admission (SAPS II and SOFA).

After adjustment, a Kaplan–Meier survival analysis was performed to determine in-ICU survival probability depending on the antibiotic treatment duration. Of note, 9 patients were excluded from the analysis due to missing data. A sensitivity analysis was performed defining SAT as a duration of antibiotic treatment of ≤10 days.

## 5. Conclusions

In our cohort, one quarter of patients with ESBL-E BSI acquired in the ICU surviving beyond 7 days were treated with a SAT. Short duration of antibiotic therapy did not appear to affect survival in patients with ESBL-E BSI acquired in the ICU. The authors recommend the prescription of SAT in the ICU patients with ESBL-E BSI regardless of the source of infection or initial severity. Exceptions should be inappropriate control of the source of infection or at-risk patients, such as transplanted patients or neutropenic patients. Additional studies are needed to assess the best duration of treatment for these specific populations.

## Figures and Tables

**Table 1:** Demographic and clinical characteristics of our patients.

Characteristics	Patients *n* = 379
Male sex	250 (66%)
Age (years)	61 (51–70)
McCabe ≥ 2	191 (50%)
SOFA score	6 (3–9)
Comorbidities	
Diabetes	106 (28%)
COPD and respiratory insufficiency	82 (22%)
Renal insufficiency	39 (10%)
Solid cancer	44 (12%)
Hematological malignancy	29 (8%)
Immunodeficiency	
Immunosuppressive therapy < 3 months	40 (11%)
Transplantation	22 (6%)
Neutropenia	13 (3%)
Medical admission	317 (84%)
Source:	
Respiratory	212 (56%)
Catheter related infection	102 (27%)
Intra-abdominal	47 (12%)
Urinary tract	20 (5%)
Microorganisms:	
*Klebsiella* sp.	169 (45%)
*Enterobacter* sp.	82 (22%)
*Escherichia coli*	37 (10%)
*Serratia marcescens*	8 (2%)
Complications	
Septic shock	169 (45%)
Acute respiratory distress syndrome	41 (29%)
Acute renal failure	85 (22%)
Disseminated intravascular coagulation	36 (9%)
Fungemia	26 (7%)
Clostridium difficile colitis	5 (1%)
Colonization with multidrug-resistant bacteria	98 (26%)
Colonization with carbapenem-resistant bacteria	24 (6%)
Outcome	
Relapse	52 (14%)
In-ICU mortality	185 (49%)

**Table 2:** Bivariate analysis comparing SAT (≤7-day antibiotic treatment) and LAT (>7-day antibiotic treatment) groups in the entire cohort and in survivors beyond 7 days.

Characteristics	Total Population			Survivors Beyond 7 Days		
	SAT *n* = 153	LAT *n* = 226	*p*	SAT *n* = 74	LAT *n* = 174	*p*
Age	62 (53–71)	60 (49–69)	0.59	61 (50–69)	60 (49–69)	0.640
Male gender	105 (69%)	145 (64%)	0.37	55 (74%)	143 (64%)	0.10
Mc Cabe ≥ 2	86 (56%)	105 (46%)	0.063	33 (45%)	105 (47%)	0.73
SOFA at admission	8 (5–11)	6 (4–10)	<0.05	8 (4–10)	6 (4–10)	0.086
Comorbidities						
Diabetes	43 (28%)	63 (28%)	0.96	19 (26%)	63 (28%)	0.68
COPD, respiratory insufficiency	26 (17%)	56 (25%)	0.071	12 (16%)	55 (25%)	0.14
Dhronic renal insufficiency	15 (9.8%)	24 (11%)	0.80	2 (3%)	24 (11%)	<0.05
Solid cancer	12 (7.8%)	32 (14%)	0.060	4 (5%)	32 (14%)	<0.05
Malignant hemopathy	18 (12%)	11 (5%)	<0.05	2 (3%)	11 (5%)	0.53
Immunodeficiency						
Immunosuppressive therapy < 3 months	14 (9%)	26 (12%)	0.46	12 (16%)	14 (6%)	0.58
Transplantation	8 (5%)	14 (6%)	0.69	0 (0)	14 (6%)	<0.05
Neutropenia	8 (5%)	5 (2%)	0.11	0 (0)	5 (2%)	0.34
Carbapenem < 3 months	40 (26%)	72 (32%)	0.25	12 (16%)	71 (32%)	<0.05
Duration of ICU stay before BSI	15 (10–26)	19 (12–30)	<0.05	15 (10–25)	19 (12–32)	<0.05
SOFA at BSI onset	7 (4–11)	5 (3–8)	<0.05	5 (2–7)	5 (3–8)	0.49
qPitt score at BSI onset	4 (2–6)	3 (2–4)	<0.05	3 (0–5)	3 (2–4)	0.37
Septic shock at BSI onset	77 (50%)	90 (40%)	<0.05	18 (24%)	89 (40%)	<0.05
Source of BSI						
Pneumonia	79 (52%)	133 (59%)	0.17	29 (39%)	133 (59%)	<0.05
Catheter related	38 (25%)	64 (28%)	0.45	28 (38%)	64 (29%)	0.13
Intra-abdominal	21 (14%)	26 (12%)	0.52	5 (7%)	25 (11%)	0.28
Urinary tract	9 (6%)	11 (5%)	0.66	3 (4%)	11 (5%)	>0.99
Secondary localization	2 (1%)	13 (6%)	0.029	2 (3%)	13 (6%)	0.37
Antibiotic treatment						
Appropriate initial treatment	105 (69%)	188 (83%)	<0.05	46 (62%)	187 (83%)	<0.05
Duration of appropriate treatment	4 (2–7)	14 (10–15)	<0.05	7 (4–7)	14 (10–15)	<0.05
Evolution						
Amines > 48 h	46 (31%)	74 (33%)	0.59	17 (23%)	73 (33%)	0.11
Duration of mechanical ventilation	4 (1–9)	14 (7–31)	<0.05	7 (0–17)	14 (7–31)	<0.05
Relapse	7 (5%)	45 (20%)	<0.05	7 (10%)	45 (26%)	<0.05
Death in ICU	105 (69%)	80 (35%)	<0.05	26 (35%)	79 (35%)	0.98
*C. difficile* colitis	0 (0)	5 (2%)	0.086	0 (0)	5 (3%)	0.34
Fungemia	4 (3%)	22 (10%)	<0.05	3 (4%)	18 (8%)	0.25
Colonization with MDR bacteria	18 (12%)	80 (36%)	<0.05	12 (16%)	79 (45%)	<0.05

## Data Availability

Data are available under request by email to the corresponding author.
